# Team sport as a potential context for interpersonal emotion regulation in early adolescence: a quasi-experimental comparison with individual exercise

**DOI:** 10.3389/fpsyt.2026.1948213

**Published:** 2026-10-01

**Authors:** Xilin Li, Qiuping You, Yunfei Tao, Chengbo Yang, Dawei Yu

**Affiliations:** 1 School of Sport Training, Chengdu Sport University, Chengdu, China; 2 School of Teacher Education (Physical Education), Taizhou University, Taizhou, China

**Keywords:** cognitive reappraisal, early adolescents, expressive suppression, individual exercise, interpersonal emotion regulation, team exercise

## Abstract

**Objective:**

Emotion regulation deficits are a transdiagnostic risk factor for adolescent emotional disorders. This quasi-experimental study examined whether different exercise contexts were associated with intrapersonal and interpersonal emotion regulation in early adolescents.

**Methods:**

A quasi-experimental cluster-allocated design was conducted with 114 sixth-grade students from three intact classes, with each class allocated to one of three conditions: team exercise, individual exercise, or control condition. Participants completed measures of emotion regulation at pre-test, mid-test, and post-test during an 8-week intervention.

**Results:**

Team exercise was associated with a significantly different trajectory of interpersonal emotion regulation over time compared with individual exercise and control conditions, with the team exercise group showing a more favorable pattern of change. Changes in expressive suppression showed a small negative association with changes in interpersonal emotion regulation.

**Conclusion:**

Team-based cooperative exercise may provide a potentially relevant context for supporting interpersonal emotion regulation in early adolescents, although the association involving expressive suppression should be interpreted cautiously given the low reliability of this subscale.

## 1 Introduction

Emotion regulation refers to individuals’ capacity to monitor, evaluate, and modulate their emotional responses across different stages of the emotion-generative process (1), encompassing both conscious and automatic regulatory efforts. It is widely recognized as a foundational psychological mechanism underlying mental health, social functioning, and adaptive behavior, and a transdiagnostic process influencing a broad range of psychological outcomes (2). A substantial body of research has demonstrated that effective emotion regulation is associated with greater psychological well-being, more satisfying interpersonal relationships, and enhanced social competence, whereas deficits in emotion regulation are linked to a wide range of internalizing and externalizing problems (3). As such, emotion regulation has been increasingly recognized as a core component of socio-emotional development across the lifespan, as it is progressively acquired and refined through developmental processes from childhood to adulthood (4).

Although traditional research has primarily conceptualized emotion regulation as an intrapersonal process—focusing on how individuals regulate their own emotions through internal cognitive and behavioral mechanisms (5)—emerging perspectives increasingly emphasize the inherently social nature of emotional processes (6). Zaki and Williams (7) introduced the concept of interpersonal emotion regulation, which refers to the processes through which individuals regulate their emotions via interactions with others, a framework that has been further elaborated in recent research (8). These processes may involve seeking social support, sharing emotional experiences, or influencing others’ emotional states in ways that subsequently alter one’s own emotions (9). Accumulating evidence suggests that interpersonal emotion regulation plays a particularly salient role during adolescence, a developmental stage characterized by a shift in social orientation from family to peers (10). In peer contexts, adolescents not only experience emotions but also learn how to understand, express, and regulate them through ongoing social interactions (11). Consequently, examining emotion regulation from both intrapersonal and interpersonal perspectives may provide a more comprehensive account of adolescents’ socio-emotional development.

Early adolescence represents a particularly sensitive period for the development of emotion regulation capacities, typically ranging from approximately 10 to 13 years of age (12, 13). Rapid cognitive and social development during this stage increases regulatory demands. While improvements in cognitive control may support intrapersonal strategies such as cognitive reappraisal, increasing orientation toward peers exposes young adolescents to more frequent and complex interpersonal emotional contexts in daily life (14). Children around 11 years of age are also transitioning from middle childhood into adolescence, when peer relationships and social-emotional competencies become increasingly important, while emotion regulation and social-cognitive capacities remain under development (15, 16). Thus, socially interactive exercise may be particularly relevant during this developmental period because team-based activities provide repeated opportunities to coordinate with peers, respond to others’ emotional and behavioral cues, and regulate one’s own emotions within shared social situations (11). Although the present study does not directly compare different age groups, these developmental characteristics suggest that early adolescence may represent an important period in which socially interactive contexts could support the development and practice of interpersonal emotion regulation. Difficulties in emotion regulation during this period have been associated with later psychological maladjustment, highlighting the importance of contextual factors that may support adaptive development (17).

Physical activity has been widely recognized as an important environmental and behavioral context that contributes to adolescents’ psychological well-being. A growing body of literature has demonstrated that regular participation in physical activity is associated with reduced symptoms of anxiety and depression, improved mood states, and enhanced self-esteem and social functioning (18). However, evidence for these broad mental health benefits should be distinguished from evidence specifically addressing emotion regulation. Although systematic reviews and meta-analyses have reported positive associations between sport participation and psychosocial outcomes such as emotional competence and interpersonal skills (19, 20), these outcomes do not necessarily capture how adolescents regulate their own emotions or regulate emotions within interpersonal contexts. Research directly examining emotion regulation in relation to physical activity has increasingly addressed these processes, but the specific role of exercise context in interpersonal emotion regulation remains less well understood (21, 22). Beyond its general psychological benefits, physical activity provides a structured context in which adolescents can experience emotionally salient situations, practice coping strategies, and engage in meaningful social interactions (23). These characteristics suggest that physical activity may provide opportunities for emotion-regulation processes to occur, particularly within socially interactive settings.

Importantly, recent research has moved beyond broad categorizations of physical activity and increasingly emphasizes the need to specify the structural and interactional features that differentiate exercise modalities (24). In this context, the distinction between team-based and individual-based exercise is not merely based on the number of participants, but rather on the extent to which the activity involves interdependence, shared goals, coordinated action, and reciprocal social interaction. In the present study, team exercise is conceptualized as a form of physical activity characterized by high levels of task interdependence, real-time communication, and collective goal pursuit, in which individual performance is inherently embedded within group dynamics. In contrast, individual exercise is defined as activity primarily structured around self-paced performance, independent task execution, and minimal reliance on peer interaction, even when performed in a shared physical space (25). This distinction is theoretically meaningful because these structural features are likely to shape the emotional demands and regulatory processes experienced during activity. Specifically, team-based contexts may provide more opportunities for individuals to monitor both their own and others’ emotional states and to engage in emotionally relevant social interactions. By contrast, individual exercise contexts may place greater emphasis on intrapersonal regulation with relatively fewer opportunities for socially mediated emotional exchange (26).

At the same time, the distinction between team-based and individual-based exercise is necessarily intertwined with other characteristics of the specific activities used to operationalize these conditions. In the present study, balloon volleyball and badminton differ not only in their degree of social interaction and task interdependence, but potentially also in motor demands, skill-acquisition requirements, competitive structure, perceived novelty and enjoyment, perceived competence, and instructor–participant interactions. These characteristics may independently influence participants’ emotional experiences and regulation during exercise. Therefore, the present design does not permit the effects of social interactional context to be isolated completely from the broader characteristics of exercise modality. Rather, the team and individual conditions should be understood as representing different exercise contexts in which social interaction and task interdependence constitute theoretically important, but not exclusive, distinguishing features.

Accordingly, differences observed between the two exercise conditions should not be interpreted as evidence that social interaction alone accounts for differences in emotion regulation. Instead, the present study examines whether exercise contexts that differ systematically in their social interaction and task-interdependence characteristics are associated with different patterns of interpersonal and intrapersonal emotion regulation.

Although previous intervention studies have demonstrated that structured physical activity can influence adolescents’ psychological and emotional outcomes, they have generally focused on the overall effects of exercise or broad psychological outcomes rather than examining whether different exercise contexts are associated with changes in interpersonal emotion regulation (27). At the same time, observational and sport-based research has increasingly examined the interpersonal and social dimensions of emotion regulation, including the regulation of emotions between athletes, teammates, and coaches (28). However, such observational evidence does not establish whether different exercise contexts are accompanied by differential changes in emotion regulation over time. Thus, it remains unclear whether exercise contexts characterized by different levels of social interaction and task interdependence are associated with different patterns of change in interpersonal emotion regulation when compared across team-based exercise, individual exercise, and a control condition.

Accordingly, the present study examined whether distinct exercise contexts (team-based vs. individual-based exercise) were differentially associated with interpersonal and intrapersonal emotion regulation in early adolescents. The study focused on interpersonal emotion regulation as the primary outcome, while cognitive reappraisal and expressive suppression were examined as complementary intrapersonal emotion-regulation dimensions. Rather than assuming a uniform relationship between exercise and emotion regulation or attributing potential differences solely to social interaction, the study sought to describe and compare the trajectories of interpersonal emotion regulation and whether changes in intrapersonal emotion-regulation strategies were related to changes in interpersonal emotion regulation. The conceptual framework underlying the present study is presented in **Figure 1**.

**Figure 1:**
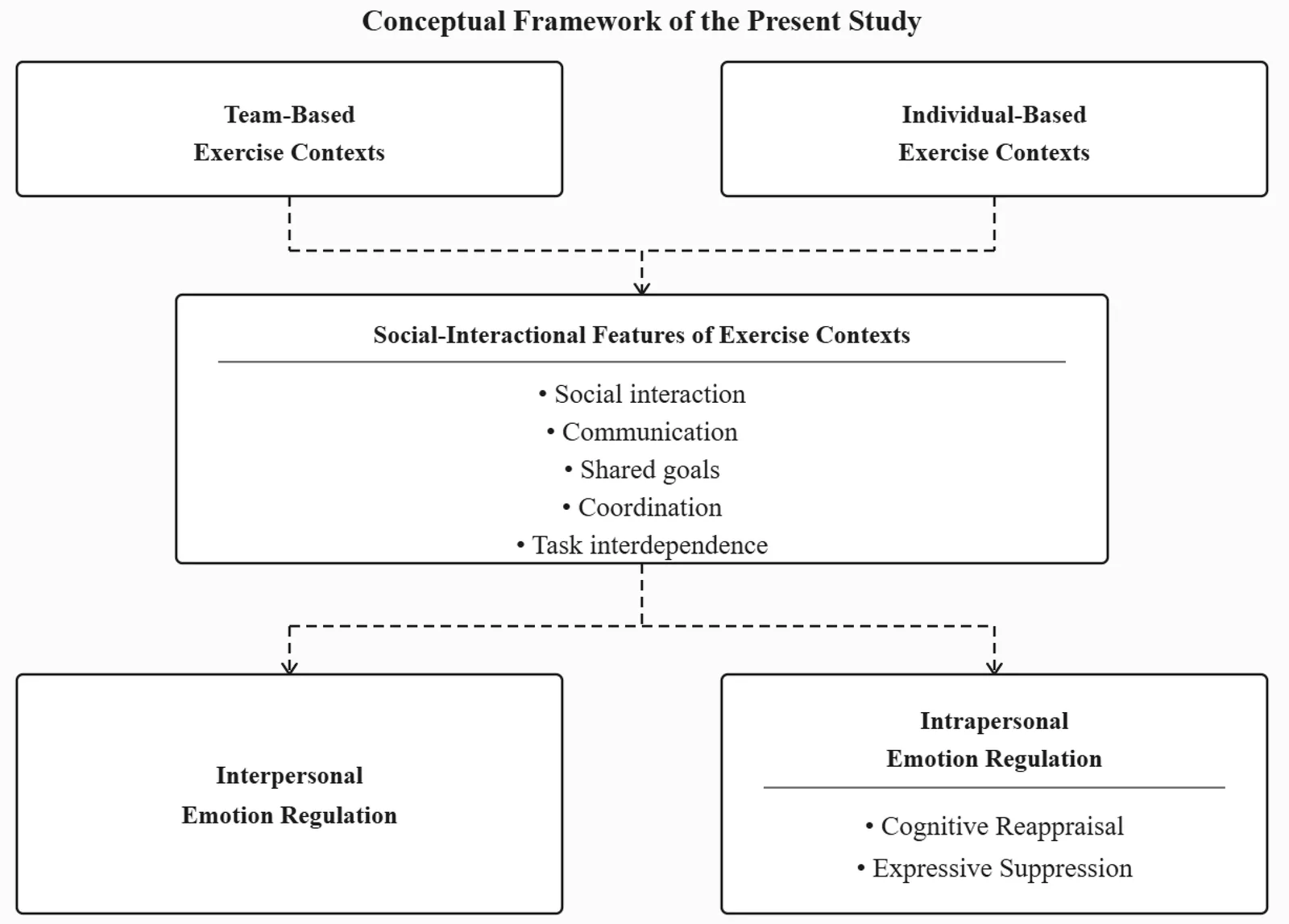
Conceptual framework underlying the present study.

Based on the theoretical framework and previous literature, three hypotheses were proposed. First, participants in the team-based exercise condition were expected to show greater improvements in interpersonal emotion regulation than those in the individual-based exercise and control conditions (H1). Second, greater improvements in cognitive reappraisal were expected to be positively associated with greater improvements in interpersonal emotion regulation (H2). Third, greater increases in expressive suppression were expected to be negatively associated with changes in interpersonal emotion regulation (H3).

## 2 Methods

### 2.1 Participants

An *a priori* power analysis was conducted using G*Power. With a two-sided significance level of α=0.05, the analysis indicated that a minimum of 67 participants was required. Allowing for an anticipated 10% attrition rate, the target recruitment sample was set at 75 participants. Accordingly, a total of 120 sixth-grade students from three intact classes in a primary school in China were initially recruited to participate in the study (40 students per class). Because the intervention was implemented within existing school classes, intact classes, rather than individual students, served as the unit of allocation. The three classes were allocated to the team-based exercise, individual-based exercise, or control condition using a computer-generated randomization procedure prior to the intervention. This class-level allocation was adopted to avoid contamination between intervention conditions and to maintain the normal organization of school teaching activities. Given that only three classes served as clusters, however, the allocation procedure could not ensure adequate balance in class-level characteristics across conditions. Because each class was assigned to a single condition, intervention condition was completely confounded with class membership. Thus, although random allocation was used to reduce systematic allocation bias, the present design cannot completely separate intervention effects from pre-existing class-level characteristics, such as classroom climate, peer dynamics, or other class-specific factors.

All three classes were taught by the same physical education (PE) instructor throughout the intervention period. The instructor was not informed of the specific research hypotheses concerning the expected differences between the team-based, individual-based, and control conditions. Thus, both exercise interventions were delivered by the same instructor, and awareness of the hypothesized direction of the intervention effects was minimized.

Participants were excluded from the final analyses if they (a) failed to complete either the pre-test or post-test assessment, (b) had substantial missing questionnaire data, or (c) attended fewer than two-thirds of the intervention sessions. To identify potentially invalid responses, response patterns were examined for clear signs of inattentive responding. Specifically, highly repetitive response patterns were identified when participants selected the same response option across a long sequence of items or followed an obvious repetitive response pattern, such as repeatedly selecting response options in a fixed sequence (e.g., 1–2–3–4). Response consistency was also checked by examining responses to reverse-scored items for obvious contradictions. To minimize invalid responses, questionnaires were reviewed immediately after completion. When obvious response abnormalities or signs of inattentive responding were identified during questionnaire administration, participants were given an opportunity to complete the questionnaire again. During subsequent data entry and verification, questionnaires were further examined for response-pattern abnormalities.

Of the 120 participants initially recruited, one participant from the individual-exercise group was excluded because attendance fell below two-thirds of the intervention sessions. In addition, five participants were excluded during subsequent data entry and verification because their questionnaires showed clear highly repetitive response patterns, including three participants from the team-sport group and two participants from the control group. These five participants were not asked to complete the questionnaire again because the problematic response patterns were identified during subsequent data verification rather than during questionnaire administration. The final analytic sample consisted of 114 participants, including 37 participants in the team-sport group, 39 participants in the individual-exercise group, and 38 participants in the control group (**Figure 2**). Overall, 95.0% of the initially recruited participants were retained in the final analytic sample. The gender and age distribution of the three groups of participants is shown in **Table 1**.

**Figure 2:**
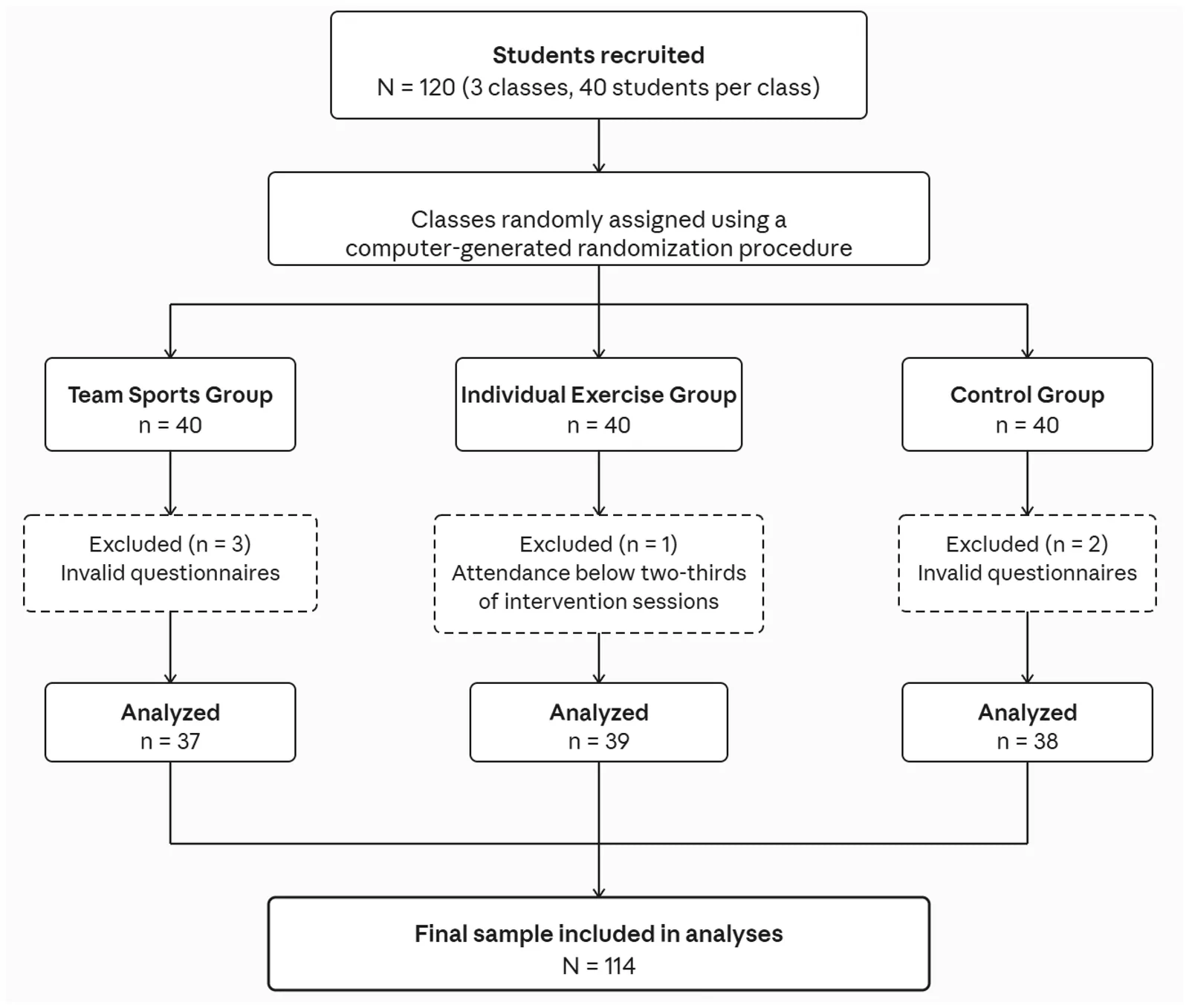
Flow diagram of participant recruitment, allocation, exclusion, and final inclusion in the analyses.

**Table 1:** Demographic variable description.

Variable	Team sports group (n=37)	Individual exercise group (n=39)	Control group (n=38)
Gender (Male/Female)	20/17	21/18	22/16
Average Age (Years, M ± SD)	11.32 ± 0.53	11.28 ± 0.51	11.32 ± 0.47

The study protocol was reviewed and approved by the Ethics Committee of Chengdu Sport University (Approval No.2025215), and all procedures were conducted in accordance with the Declaration of Helsinki. Written informed consent was obtained from all participating students and their parents or legal guardians prior to the commencement of the study.

### 2.2 Study design and procedure

This study employed a quasi-experimental, pretest–midtest–posttest controlled design with three parallel groups: a team exercise group, an individual exercise group, and a control group. The intervention was conducted in a real school setting over 8 weeks. Because the intervention was implemented within existing school classes, the three intact classes were allocated to the three conditions at the class level using a computer-generated randomization procedure prior to the intervention. While individual random allocation was not feasible due to the school setting, class-level allocation allowed the intervention to be implemented within the existing school structure while reducing potential contamination between conditions. This design allowed for the examination of changes in emotion regulation over time while preserving ecological validity in a naturalistic school-based intervention environment. Baseline characteristics were compared across the three groups before the main analyses.

The intervention lasted eight weeks with two 40-minute sessions per week, and all exercise sessions followed a standardized structure: 5-minute warm-up, 30-minute main activities, and 5-minute cool-down. All sessions were delivered by trained physical education instructors according to a standardized protocol to ensure consistency. Moderate intensity was adopted for the intervention, comprehensively considering participants’ developmental characteristics and the nature of study variables—this intensity sustains stable psychological arousal, ensures physical safety, and facilitates cognitive engagement and emotional processing during activity (29). Exercise intensity was monitored using the Polar Team Pro heart rate monitoring system during the exercise sessions for the team and individual groups and during regular PE classes for the control group. For each class, six students were randomly selected for heart rate monitoring, and the same six students were monitored throughout the intervention. Heart rate was recorded continuously from the beginning to the end of each exercise session. This monitoring was used to verify that the exercise intensity was consistent with the intended moderate-intensity level. Attendance was recorded throughout to ensure compliance.

To enhance conceptual clarity, the two exercise conditions were systematically structured to reflect distinct social-interactional and task-related characteristics associated with team-based and individual-based activities, while keeping exercise intensity, duration, and instructional format consistent across conditions. Specifically, the team exercise group engaged in volleyball-based activities designed to emphasize high levels of task interdependence, real-time communication, and collective goal pursuit. The activities included cooperative drills, sequential passing tasks, and group-based challenges that required continuous coordination and mutual adjustment among participants. In these tasks, successful performance depended on collective execution rather than individual proficiency, thereby embedding individual behavior within a dynamic social context. In contrast, the individual exercise group participated in badminton-based activities structured to emphasize independent skill execution, self-paced practice, and minimal task interdependence. Although participants performed activities within the same physical environment, tasks were designed to reduce the necessity for coordination or communication with peers. Performance outcomes were primarily determined by individual skill development and personal effort rather than group collaboration (**Table 2**). Thus, the two conditions represented distinct exercise contexts that differed in their social-interactional and task-related characteristics. Because these characteristics were operationalized through different sports, the team-based and individual-based conditions cannot be interpreted as differing only in social structure; rather, they reflect the broader characteristics of the respective exercise contexts. The control group received no additional intervention and only attended regular school physical education classes.

**Table 2:** Specific intervention content of team exercise and individual exercise groups.

Week	Team exercise group (volleyball)	Individual exercise group (badminton)
1	Familiar with the ball, practice underhand serve	Familiar with the ball, practice the ball
2	Review underhand serve, learn double ground pass catch the ball	Review the ball, learn to serve
3	Review the ground pass, learn multiplayer sequential pass	Review serve, learn fixed point swing hit the ball
4	Review multiplayer pass, learn double mat ball cooperation	Review swing batting, learn to move swing batting
5	Review double mat ball, learn three fixed-point pass mat ball	Review swing batting, learn the basic pace
6	Review three pass, learn six fixed-point pass cushion ball	Review the basic pace, learn the combination of pace and batting
7	Repeated practice six fixed-point pass cushion ball	Repeatedly practice hitting the ball in the moving pace
8	Group cycle challenge	Single cycle challenge

Psychological measures were administered at three time points: baseline (pre-test), mid-intervention (week 4), and post-intervention (week 8).

### 2.3 Measures

#### 2.3.1 Intrapersonal emotion regulation

Intrapersonal emotion regulation was assessed using the Chinese version of the Emotion Regulation Questionnaire for Children and Adolescents (ERQ-CA-C). The original Emotion Regulation Questionnaire was developed by Gross and John (30), and the Chinese version for children and adolescents was adapted and validated by Chinese researchers (31).

The scale consists of 10 items and includes two subscales: cognitive reappraisal and expressive suppression. All items are rated on a five-point Likert scale, with higher scores indicating greater use of the corresponding emotion regulation strategy.

In the present study, the Cronbach’s α coefficients for the cognitive reappraisal and expressive suppression subscales were 0.711 and 0.567, respectively. The internal consistency of the expressive suppression subscale was relatively low (*α* = 0.567), indicating limited reliability in the present sample. Therefore, findings involving expressive suppression, particularly nonsignificant results and associations with other variables, should be interpreted with caution.

A Kaiser–Meyer–Olkin (KMO) value of 0.643 and a significant Bartlett’s test of sphericity (*χ²* = 193.707, *p* <.001) indicated that the correlation matrix was suitable for exploratory analysis. As an additional exploratory check of the factor structure of the ERQ-CA-C in the present Chinese early-adolescent sample, a principal component analysis (PCA) was conducted. This analysis was intended as an exploratory, sample-specific check rather than a confirmatory validation of the previously established factor structure of the ERQ-CA-C. Using PCA with Varimax rotation and Kaiser normalization, two components were extracted, accounting for 43.51% of the total variance. The resulting two-component structure was broadly consistent with the expected dimensions of cognitive reappraisal and expressive suppression. Given the relatively low proportion of variance explained, these findings should be interpreted cautiously.

#### 2.3.2 Interpersonal emotion regulation

Interpersonal emotion regulation was measured using the Interpersonal Regulation Questionnaire (IRQ) originally developed by Williams and colleagues (32) and later adapted into Chinese (Interpersonal Emotion Regulation Questionnaire, IERQ) by Liao Longyue and colleagues (33).

The scale contains 16 items across four dimensions: negative emotion tendencies, negative emotion efficacies, positive emotion tendencies, and positive emotion efficacies. Items are rated on a seven-point Likert scale ranging from 1 (strongly disagree) to 7 (strongly agree). Mean scores are calculated for each dimension, with higher scores indicating higher levels of interpersonal emotion regulation.

The scale assesses two key aspects of interpersonal emotion regulation: individuals’ tendency to seek others’ assistance in regulating emotions and their perceived efficacy of such regulation, in both negative and positive emotional contexts.

In the present sample, the scale demonstrated good internal consistency, with a Cronbach’s α coefficient of 0.798. As an additional exploratory check of the factor structure of the IERQ in the present sample, a principal component analysis (PCA) was conducted. A Kaiser–Meyer–Olkin (KMO) value of 0.843 and a significant Bartlett’s test of sphericity (*χ²* = 675.063, df=120, *p* <.001) indicated that the correlation matrix was suitable for exploratory analysis. Given the sample size and the scale structure, the factor-analytic findings were interpreted cautiously. Using PCA with Varimax rotation and Kaiser normalization, four components were extracted, accounting for 60.56% of the total variance. The resulting four-component structure was broadly consistent with the expected dimensions of interpersonal emotion regulation.

### 2.4 Statistical analysis

All statistical analyses were conducted using IBM SPSS Statistics. Descriptive statistics, including means and standard deviations, were first calculated for all study variables.

To address the primary research question, a 3 (group) × 3 (time) mixed-design ANOVA was conducted. The between-subjects factor was group (team exercise, individual exercise, control), and the within-subjects factor was time (pre-test, mid-test, post-test). The assumptions relevant to the mixed-design ANOVA were assessed prior to interpretation of the results. Homogeneity of variance was examined using Levene’s test. For the within-subjects factor, Mauchly’s test of sphericity was conducted. When the sphericity assumption was violated, Greenhouse–Geisser corrections were applied to the degrees of freedom. The distributions of the outcome variables were also inspected for substantial deviations from normality.

Because the intervention was allocated at the intact-class level, students were nested within three classes, with one class representing each study condition. Given the very small number of level-2 units, multilevel modelling and cluster-robust estimation were not considered statistically reliable. The inferential analyses were therefore conducted at the individual-student level.

To further examine between-group differences in outcome scores while controlling for baseline levels, supplementary one-way ANCOVAs were conducted using the corresponding pre-test score as the covariate. For interpersonal emotion regulation, separate ANCOVAs were conducted at mid-test and post-test, with pre-test interpersonal emotion regulation entered as the covariate. For cognitive reappraisal, an ANCOVA was conducted at post-test, with pre-test cognitive reappraisal entered as the covariate.

Secondary analyses were conducted to examine intrapersonal emotion regulation outcomes. The same mixed-design ANOVA procedure was applied separately to cognitive reappraisal and expressive suppression. When significant omnibus effects were detected, Bonferroni-adjusted pairwise comparisons were conducted for the relevant *post hoc* and simple-effects analyses to control the family-wise Type I error rate.

To explore potential associations between intrapersonal and interpersonal emotion regulation processes, Pearson correlation analyses were conducted based on change scores (post-test minus pre-test). In addition, multiple regression analyses were performed to examine whether changes in intrapersonal emotion regulation variables were associated with changes in interpersonal emotion regulation. Variance inflation factors (VIFs) were examined to assess multicollinearity. The level of statistical significance was set at *p* <.05.

## 3 Results

### 3.1 Baseline equivalence

Prior to the intervention, a one-way ANOVA was conducted to examine potential baseline differences among the three groups in cognitive reappraisal, expressive suppression, and interpersonal emotion regulation. The results indicated that no statistically significant between-group differences were detected for any of the variables (all *p*>.05), indicating no evidence of statistically significant baseline differences among the three groups (**Table 3**).

**Table 3:** One-way ANOVA of pre-experiment cognitive reappraisal, expressive suppression and interpersonal emotion regulation.

Variable	Sum of squares	Degree of freedom	Mean square	*F*	*p*
Cognitive reappraisal	97.573	2	48.786	1.992	0.141
Expressive suppression	19.515	2	9.758	0.768	0.467
Interpersonal emotion regulation	264.485	2	132.243	2.327	0.102

In addition, exercise intensity, a key control variable in the intervention, was verified using Polar heart rate monitors to ensure intergroup consistency. The results showed that the mean exercise heart rates of the team exercise group, individual exercise group, and control group were 131 ± 12, 128 ± 15, and 127 ± 16 beats per minute (bpm), respectively. Correspondingly, the average exercise heart rate accounted for 65.5 ± 6.0%, 64.0 ± 7.5%, and 64.8 ± 7.7% of the predicted maximum heart rate for the three groups, all of which fell within the predefined moderate-intensity range. One-way ANOVA further revealed no statistically significant intergroup differences in either mean exercise heart rate or the percentage of predicted maximum heart rate (all *p*>.05). These findings indicated that no statistically detectable between-group differences in exercise intensity were observed during the respective PE sessions.

### 3.2 Descriptive statistics

The means and standard deviations of cognitive reappraisal, expressive suppression, and interpersonal emotion regulation for the three groups at pre-test, mid-test, and post-test are presented in **Table 4**.

**Table 4:** Pre-, mid-, and post-test scores of psychological variables for the three groups (M ± SD).

Variable	Measuring time	Team exercise group	Individual exercise group	Control group
Cognitive reappraisal	Pre-test	22.027 ± 5.225	20.513 ± 4.412	19.789 ± 5.189
	Mid-test	22.243 ± 5.351	20.436 ± 4.376	19.974 ± 5.673
	Post-test	23.081 ± 4.810	19.538 ± 4.946	20.079 ± 4.978
Expressive suppression	Pre-test	10.865 ± 3.824	10.718 ± 3.292	9.921 ± 3.575
	Mid-test	10.162 ± 3.304	10.359 ± 2.933	9.658 ± 3.331
	Post-test	9.405 ± 3.819	10.949 ± 3.228	10.316 ± 3.640
Interpersonal emotion regulation	Pre-test	62.270 ± 19.324	77.179 ± 12.908	74.553 ± 16.497
	Mid-test	77.135 ± 12.951	72.333 ± 12.815	71.868 ± 16.069
	Post-test	89.297 ± 12.295	69.667 ± 14.981	73.000 ± 15.392

From the descriptive results, the team exercise group showed higher cognitive reappraisal scores than the individual exercise and control groups across the assessment periods, whereas the individual exercise and control groups showed relatively similar levels. Expressive suppression remained relatively stable across all three groups.

Regarding interpersonal emotion regulation, the team exercise group showed a consistent upward trend across the assessment periods, whereas the individual exercise and control groups showed comparatively smaller changes.

### 3.3 Effects of exercise intervention on interpersonal emotion regulation

A 3 (group: team exercise, individual exercise, control) × 3 (time: pre-test, mid-test, post-test) mixed-design repeated-measures ANOVA was conducted to examine changes in interpersonal emotion regulation across the three study conditions. Mauchly’s test indicated that the assumption of sphericity was violated for the within-subjects effects; therefore, Greenhouse–Geisser corrections were applied.

The analysis revealed a significant main effect of time, *F*(1.76, 195.58)=11.083, *p* <.001, partial *η²* = 0.166. In contrast, the main effect of group was not significant, *F*(2, 111)=0.761, *p* = .469, partial *η²* = 0.014. Importantly, a significant time-by-group interaction was observed, *F*(3.52, 195.58)=34.779, *p* <.001, partial *η²* = 0.260 (**Table 5**), indicating that changes in interpersonal emotion regulation over time differed across the three conditions.

**Table 5:** Repeated-measures ANOVA results for interpersonal emotion regulation effect.

Variable	Effect	*F*	*P*	Partial*η*²
Interpersonal emotion regulation	Time	11.083	<.001	0.166
	Groups	0.761	.469	0.014
	Time × Group	34.779	<.001	0.260

Effect sizes were interpreted using conventional benchmarks, with partial η² values of 0.01, 0.06, and 0.14 representing small, medium, and large effects, respectively (34).

Descriptive statistics showed that interpersonal emotion regulation increased substantially in the team exercise group from pre-test (M = 62.270, SD = 19.324) to mid-test (M = 77.135, SD = 12.951) and post-test (M = 89.297, SD = 12.295). In contrast, scores decreased from pre-test to post-test in both the individual exercise group (from M = 77.179, SD = 12.908 to M = 69.667, SD = 14.981) and the control group (from M = 74.553, SD = 16.497 to M = 73.000, SD = 15.392). The corresponding trajectories are presented in **Figure 3**.

**Figure 3:**
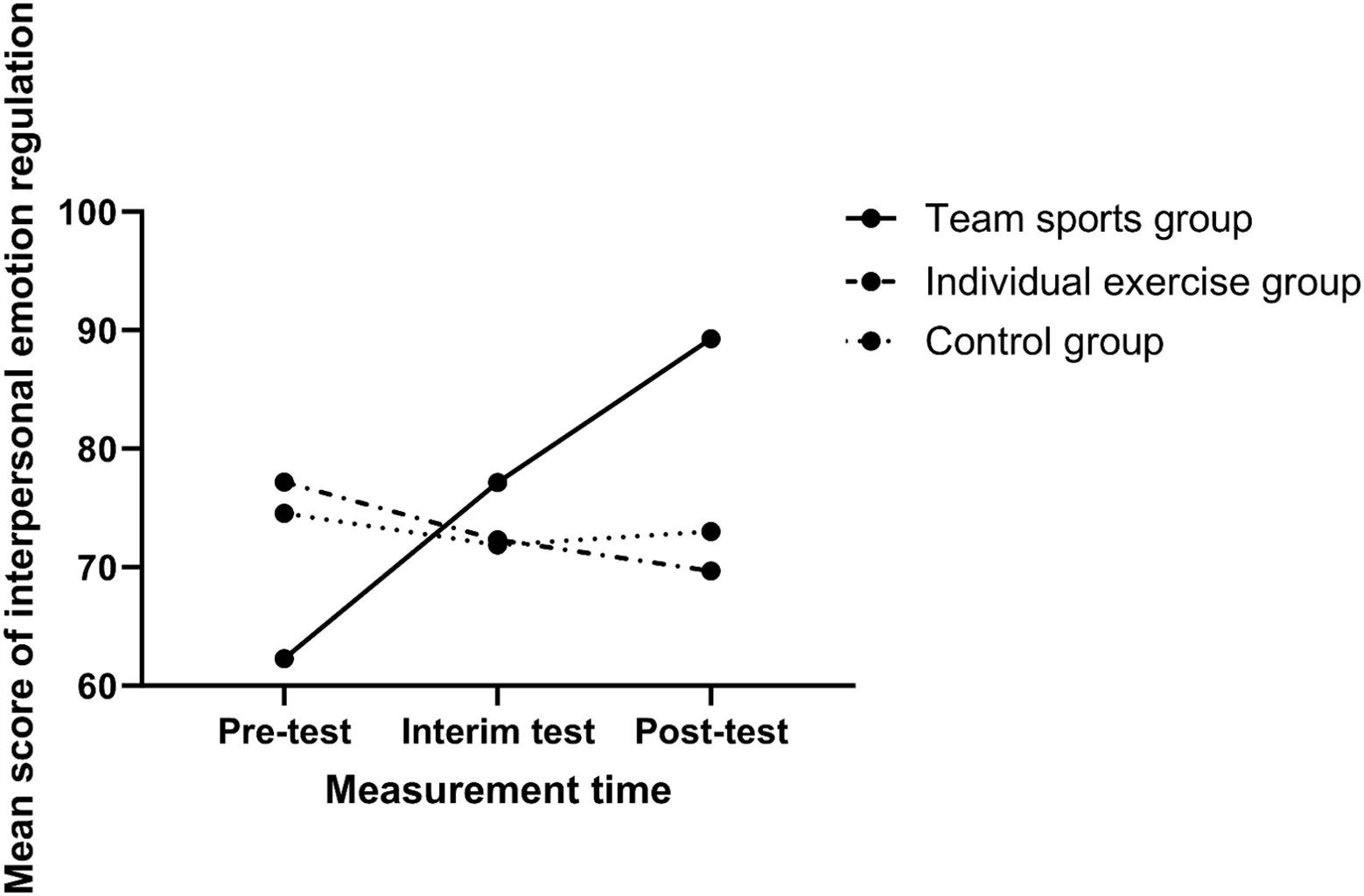
Changes in interpersonal emotion regulation scores of early adolescents before, during, and after exercise intervention.

To further examine between-group differences at the post-intervention assessments while accounting for baseline differences in interpersonal emotion regulation, supplementary ANCOVAs were conducted using pre-test interpersonal emotion regulation as a covariate. At mid-test, the adjusted group effect was significant, *F*(2, 110)=9.939, *p* <.001, partial *η²* = 0.153. The adjusted mean was 81.397 (95% CI [77.343, 85.452]) for the team exercise group, 70.437 (95% CI [66.608, 74.267]) for the individual exercise group, and 69.684 (95% CI [65.848, 73.521]) for the control group. The adjusted mean for the team exercise group was significantly higher than that of the control group (mean difference=10.960, 95% CI [5.290, 16.630], *p* <.001) and the individual exercise group (mean difference=11.713, 95% CI [5.960, 17.466], *p* <.001), whereas the individual exercise and control groups did not differ significantly (mean difference=0.753, 95% CI [−4.608, 6.113], *p* = .781).

At post-test, the adjusted group effect was also significant, *F*(2, 110)=43.959, *p* <.001, partial η²=0.444. The adjusted mean was 93.555 (95% CI [89.381, 97.728]) for the team exercise group, 71.570 (95% CI [67.629, 75.512]) for the individual exercise group, and 67.021 (95% CI [63.072, 70.970]) for the control group. The adjusted mean for the team exercise group was significantly higher than that of the control group (mean difference=21.985, 95% CI [16.148, 27.821], *p* <.001) and the individual exercise group (mean difference=26.534, 95% CI [20.613, 32.456], *p* <.001). The difference between the individual exercise and control groups was not statistically significant (mean difference=4.550, 95% CI [−0.968, 10.067], *p* = .105).

### 3.4 Cognitive reappraisal across exercise conditions

A mixed-design ANOVA was conducted as a secondary analysis to examine cognitive reappraisal across groups and time. The results indicated a significant main effect of group, *F*(2, 111)=4.267, *p=.*016, partial *η²* = 0.071, whereas the main effect of time was not significant, *F*(2, 222)=0.042, *p* = .980, partial *η²* = .000. The interaction between time and group was also not significant, *F*(4, 222)=0.950, *p* = .436, partial *η²* = 0.017.

*Post hoc* comparisons indicated that the team exercise group reported higher cognitive reappraisal scores than both the individual exercise group and the control group. However, because neither the main effect of time nor the time × group interaction was significant, these between-group differences should not be interpreted as evidence of an intervention-related increase in cognitive reappraisal. Rather, they indicate differences in overall cognitive reappraisal levels among the three groups.

An ANCOVA controlling for baseline scores showed a similar pattern, with a significant main effect of group, *F*(2, 110)=4.101, *p* = .019, partial*η²* = 0.069. Baseline cognitive reappraisal was also a significant covariate, *F*(1, 110)=38.337, *p* <.001, partial *η²* = 0.258, indicating that baseline cognitive reappraisal was significantly associated with post-intervention scores (**Table 6**).

**Table 6:** ANCOVA results for cognitive reappraisal controlling for baseline.

Source	*df*	*F*	*p*	Partial*η*²
Baseline cognitive reappraisal	1	38.337	<.001	0.258
Group	2	4.101*	.019	0.069

*p <.05.

### 3.5 Expressive suppression across exercise conditions

Expressive suppression was examined across exercise contexts and time points.

The results indicated that neither the main effect of group nor the main effect of time was significant, and the interaction between time and group was also non-significant(all *p*>.05). These findings suggest that expressive suppression remained relatively stable across the intervention period and was not significantly influenced by exercise context within the duration of the study.

### 3.6 Correlations among emotion regulation variables

Correlation analyses were conducted to examine associations between changes in intrapersonal emotion regulation (cognitive reappraisal and expressive suppression) and changes in interpersonal emotion regulation.

The results showed that changes in cognitive reappraisal were not significantly associated with changes in interpersonal emotion regulation (*r* = 0.074, *p* = .434). In contrast, changes in expressive suppression were significantly but weakly negatively correlated with changes in interpersonal emotion regulation (*r*=−0.202, *p* = .031) (**Table 7**).

**Table 7:** Correlation matrix of changes in emotion regulation variables (n=114).

Variable	Cognitive reappraisal	Expressive suppression	Interpersonal emotion regulation
Cognitive reappraisal	1		
Expressive suppression	0.322**	1	
Interpersonal emotion regulation	0.074	−0.202*	1

**p <.001, *p <.05.

To further explore whether intrapersonal emotion regulation processes were associated with changes in interpersonal emotion regulation, a multiple regression analysis was conducted. The overall regression model was statistically significant, *F*(2, 111)=3.690, *p* = .028, explaining 6.2% of the variance in changes in interpersonal emotion regulation (R²=.062; adjusted R²=.045). Changes in expressive suppression were a significant negative predictor of changes in interpersonal emotion regulation (β=−.252, *p* = .011, 95% CI [−2.279, −0.305]), whereas changes in cognitive reappraisal were not a significant predictor (β=.155, *p* = .113, 95% CI [−0.158, 1.475]) (**Table 8**). No multicollinearity issues were detected (VIFs < 2).

**Table 8:** Multiple linear regression predicting changes in interpersonal emotion regulation.

Predictor	B	SE	β	*t*	*p*	95% CI
Cognitive reappraisal	0.659	0.412	0.155	1.599	.113	[−0.158, 1.475]
Expressive suppression	−1.292	0.498	−0.252*	−2.595	.011	[−2.279, −0.305]

B = unstandardized regression coefficient; β = standardized regression coefficient; SE=standard error. *p <.05.

## 4 Discussion

The present study examined associations between distinct exercise contexts and both interpersonal and intrapersonal emotion regulation in early adolescents. The findings indicated that interpersonal emotion regulation changed differently over time across the team exercise, individual exercise, and control conditions, with the team exercise group showing the greatest increase over time. In contrast, evidence for intrapersonal emotion regulation was more limited. No significant time-related changes or time-by-group interaction effects were observed for cognitive reappraisal or expressive suppression, although significant between-group differences were observed in overall cognitive reappraisal levels. Correlational analyses further showed that changes in expressive suppression were negatively associated with changes in interpersonal emotion regulation, whereas changes in cognitive reappraisal were not significantly related to changes in interpersonal emotion regulation.

With regard to the prespecified hypotheses, the findings provided partial support for the proposed pattern. The findings were consistent with the hypothesis concerning interpersonal emotion regulation, as the team exercise group showed the greatest increase over time, whereas the hypotheses concerning cognitive reappraisal and expressive suppression were not supported, as neither outcome showed a significant time-by-group interaction. These findings suggest that associations between exercise context and emotion regulation may be more evident for interpersonal than for intrapersonal regulation.

Taken together, these findings suggest that different dimensions of emotion regulation may not be equally associated with exercise contexts during early adolescence. In the present study, the relationship between exercise context and emotion regulation was more evident for interpersonal emotion regulation than for intrapersonal regulatory strategies. Rather than suggesting a uniform relationship between exercise participation and emotion regulation, this pattern highlights the possibility that interpersonal and intrapersonal dimensions may be differentially related to the characteristics of exercise experiences. This perspective may contribute to a more nuanced understanding of how exercise contexts relate to adolescents’ socio-emotional development.

### 4.1 Team exercise and interpersonal emotion regulation

A key contribution of the present study is the finding that interpersonal emotion regulation changed differently over time across the team exercise, individual exercise, and control conditions, with participants in the team exercise condition showing the greatest increase over time. This finding highlights the potential importance of social context for adolescents’ emotion regulation processes and supports the view that emotion regulation is not solely an intrapersonal phenomenon but is fundamentally embedded in interpersonal interaction.

Interpersonal emotion regulation refers to processes through which individuals influence their own and others’ emotional states through social interaction (7). In contrast to intrapersonal strategies that operate within the individual, interpersonal emotion regulation is inherently relational and depends on ongoing exchanges with others, including emotional communication, support seeking, and affective influence.

Team exercise environments are particularly likely to provide opportunities for such processes through continuous coordination, shared goals, and emotionally salient group interactions. Within these contexts, adolescents are frequently required to respond to teammates’ emotional cues, provide encouragement, and adjust their own emotional expressions to maintain group functioning (21). Such repeated interpersonal exchanges may provide opportunities for interpersonal emotion regulation, although this possibility was not directly tested in the present study.

From a social learning perspective, interpersonal emotion regulation can be understood as a skill acquired through observation, imitation, and feedback in social contexts (35). Team exercise provides opportunities for such learning, as adolescents observe how peers and coaches respond to success, failure, and interpersonal conflict. Such experiences may be relevant to adolescents’ emotional responding in social situations (36).

In addition, theories of emotional contagion and social coordination suggest that emotions are dynamically transmitted and regulated within groups (37). In team exercise, individuals are not only influenced by others’ emotions but also actively contribute to the emotional climate of the group. This bidirectional process may be relevant to adolescents’ sensitivity to others’ emotional states and their capacity for interpersonal emotion regulation.

Furthermore, team cohesion and perceived social support have been identified as key contextual factors that facilitate adaptive emotional functioning in group settings (38). Supportive team environments may reduce emotional stress and encourage open emotional expression, and may provide a context in which interpersonal regulation processes can occur during interaction (39).

Importantly, interpersonal emotion regulation in sport contexts may also operate as a form of co-regulation, in which emotional stability emerges through reciprocal interpersonal exchanges rather than individual regulation alone (26). This perspective suggests that emotional development in adolescence is not only an internal process but also a socially distributed one that may be influenced by repeated group experiences.

Overall, the present findings suggest that team-based physical activity may provide a particularly rich context for supporting interpersonal emotion regulation during early adolescence, due to its inherent social, emotional, and cooperative demands. Importantly, this interpretation reflects an association between the team exercise context and greater changes in interpersonal emotion regulation rather than evidence that team exercise directly caused these changes.

### 4.2 Team exercise and intrapersonal emotion regulation

The results indicated that participants in the team exercise condition showed higher overall cognitive reappraisal scores than those in the individual exercise and control conditions. However, because neither the main effect of time nor the time × group interaction was significant, this between-group difference should not be interpreted as evidence that team exercise increased cognitive reappraisal over the intervention period. Instead, the finding indicates that the groups differed in their overall levels of cognitive reappraisal, with these differences showing no evidence of systematic divergence over time. Thus, the observed between-group differences may have been present before the intervention and/or remained relatively stable across the study period.

From a cognitive-developmental perspective, cognitive reappraisal is closely related to the maturation of executive functions and perspective-taking abilities during early adolescence (40). These abilities are influenced not only by biological development but also by environmental experiences (41). In this regard, team-based exercise contexts may provide structured social environments in which adolescents are exposed to diverse perspectives and evaluative feedback during interaction with peers.

Such social exposure may create opportunities for adolescents to consider alternative interpretations of emotionally salient situations, which is conceptually relevant to cognitive reappraisal processes (42). This interpretation is also consistent with social learning perspectives, which emphasize the role of observation and interaction with others in cognitive and emotional development (43).

In addition, supportive team environments characterized by cohesion and perceived social support may facilitate more flexible cognitive processing in emotionally challenging situations (39, 44). However, the present findings suggest that these contextual characteristics may primarily be reflected in between-group differences rather than short-term changes in cognitive reappraisal.

In contrast, expressive suppression showed no detectable differences in this response-focused strategy across the exercise contexts examined. As a later-stage emotion regulation strategy, expressive suppression is often considered more habitual and less sensitive to contextual variation (45). However, the relatively low internal consistency of the expressive suppression subscale in the present study (Cronbach’s α =.567) should also be considered when interpreting these findings. Measurement unreliability may have reduced the sensitivity to detect changes in expressive suppression or its associations with other variables.

### 4.3 Relationships between emotion regulation strategies

No significant association was found between changes in cognitive reappraisal and changes in interpersonal emotion regulation, contrary to the initial hypothesis. This finding suggests that changes in one regulatory domain do not necessarily correspond to changes in another regulatory domain over the study period.

The negative association between changes in interpersonal emotion regulation and changes in expressive suppression is consistent with theoretical expectations, although the magnitude of this association was small. Emotional expression plays a central role in social communication, serving as a key mechanism through which individuals signal their internal states and elicit responses from others (37). Excessive reliance on suppression may inhibit this communicative function, thereby reducing opportunities for interpersonal regulation. In team exercise contexts, where coordination and mutual understanding are essential, open emotional expression may be particularly important for effective interaction. However, this interpretation should be considered tentative, given the modest magnitude of the association and the relatively low internal consistency of the expressive suppression subscale in the present sample.

The regression analysis further indicated that changes in expressive suppression were negatively associated with changes in interpersonal emotion regulation when changes in cognitive reappraisal were considered simultaneously. However, the model explained only a modest proportion of variance (R²=.062), suggesting that changes in expressive suppression represent only one of multiple factors potentially related to changes in interpersonal emotion regulation. Although the association involving expressive suppression was statistically significant in the regression model, its relatively small effect should be interpreted cautiously. The finding therefore provides evidence of a modest statistical association rather than a strong or causal relationship between changes in expressive suppression and interpersonal emotion regulation.

Notably, the absence of a significant association between changes in cognitive reappraisal and changes in interpersonal emotion regulation suggests that these dimensions may represent relatively distinct aspects of emotional functioning. Whereas cognitive reappraisal primarily involves internal cognitive processes, interpersonal emotion regulation is embedded within social interaction and relationship dynamics. Their development may therefore depend on different mechanisms and contextual influences. This finding highlights the importance of adopting a multidimensional perspective on emotion regulation rather than assuming that improvements in one domain will automatically generalize to another.

### 4.4 Theoretical and practical implications

Theoretically, the present findings contribute to a more nuanced understanding of how emotion regulation is examined within exercise and sport contexts. Although previous research has increasingly distinguished between interpersonal and intrapersonal forms of emotion regulation (8), these dimensions are often discussed collectively when considering the psychological correlates of physical activity. The present findings suggest that they may not necessarily exhibit similar patterns of association within exercise contexts. More specifically, differences in change trajectories across exercise conditions were more evident for interpersonal emotion regulation than for cognitive reappraisal or expressive suppression, indicating that different dimensions of emotion regulation may warrant separate consideration when evaluating psychosocial outcomes of physical activity.

In particular, the findings add to a growing body of work suggesting that characteristics of exercise experiences may be relevant when considering psychological outcomes of participation (24). Rather than indicating a uniform association between exercise and emotion regulation, the results suggest that exercise contexts characterized by greater opportunities for social interaction, coordination, and shared activity engagement may be particularly relevant when considering interpersonal regulatory processes. This interpretation is broadly consistent with previous research emphasizing the social and relational features of team-based physical activity (46).

From a practical perspective, these findings have implications for school-based physical education and youth intervention programs. Incorporating structured team-based activities into school curricula may provide accessible opportunities to support adolescents’ socio-emotional development. Environments that emphasize cooperation, communication, and peer interaction may provide opportunities for naturally occurring interpersonal emotional processes during physical activity. Such approaches align with calls for more socially embedded interventions in educational settings that move beyond purely individual-focused skill development (20).

The present findings also suggest that different dimensions of emotion regulation may show different patterns of change across short-term exercise contexts. Whereas interpersonal emotion regulation showed a clearer pattern of association with participation in team-based exercise, comparable changes were not observed for cognitive reappraisal or expressive suppression. Although the present study was not designed to examine underlying mechanisms directly, this pattern highlights the value of examining interpersonal and intrapersonal emotion regulation separately when investigating psychological outcomes of physical activity. Future research should explore whether similar patterns emerge across different exercise settings, age groups, and intervention designs.

Overall, the present findings suggest that distinguishing between interpersonal and intrapersonal dimensions of emotion regulation may provide a more refined understanding of how exercise participation relates to adolescent socio-emotional development.

### 4.5 Limitations and future directions

Despite its contributions, the present study has several limitations that should be acknowledged.

First, the study used a quasi-experimental design in which participants were allocated to conditions at the intact-class level, with one class assigned to each condition. Therefore, the observed between-condition differences may be partly influenced by class-level characteristics, and the association between exercise context and the observed outcomes cannot be statistically separated from potential class effects. Although baseline characteristics were examined and supplementary ANCOVA analyses were conducted to adjust for baseline interpersonal emotion regulation, these approaches cannot fully eliminate class-level confounding. In addition, although the baseline ANOVA did not show a statistically significant group difference in interpersonal emotion regulation, the team exercise group had a lower descriptive baseline level than the other two groups. This initial numerical imbalance may have contributed to the subsequent improvement, and regression toward the mean or a natural catch-up trajectory cannot be ruled out. Therefore, the observed increase in the team exercise group should be interpreted cautiously rather than attributed solely to the exercise condition. Future studies should include multiple classes within each condition and, where feasible, use multilevel or cluster-based analytical approaches to more appropriately account for the nested structure of the data.

Second, measurement limitations should also be considered. The study relied primarily on self-report measures, which may be subject to social desirability and recall biases, particularly for socially embedded constructs such as interpersonal emotion regulation. The psychometric findings for the ERQ-CA-C warrant cautious interpretation. Although the exploratory PCA broadly reproduced the expected two-component structure, the two components accounted for 43.51% of the total variance, and the internal consistency of the expressive suppression subscale was relatively modest (Cronbach’s α = .567). The PCA was intended as a sample-specific exploratory check rather than a formal validation of the established scale structure. Future research should further examine the measurement properties of the ERQ-CA-C in larger and more diverse adolescent samples, preferably using confirmatory factor analysis when formally evaluating its established factor structure, and should incorporate multiple methods of assessment where feasible.

Third, the intervention duration was limited to 8 weeks. Although differences in change trajectories were observed over this period, longer-term longitudinal designs are needed to examine the stability and developmental trajectories of these observed changes across adolescence.

Finally, the present study did not directly examine the mechanisms underlying the association between team-based physical activity and interpersonal emotion regulation. Future research should incorporate theoretically informed variables, such as perceived social support, team cohesion, and peer interaction quality, to examine whether these factors help explain differences in interpersonal emotion regulation across exercise contexts.

## 5 Conclusions

The present study examined associations between distinct exercise contexts and emotion regulation in early adolescents. Interpersonal emotion regulation showed different patterns of change over time across the exercise conditions, with the team exercise group showing the greatest increase over time, whereas evidence for intrapersonal emotion regulation was more limited. These preliminary findings suggest that socially interactive team-exercise contexts may be associated with more favorable changes in interpersonal emotion regulation during early adolescence. However, given the limited number of clusters and class-level allocation, these findings should not be interpreted as evidence of a causal intervention effect. Further adequately powered cluster-randomized studies with multiple classes per condition are needed to establish whether such associations reflect causal effects.

## Data Availability

The raw data supporting the conclusions of this article will be made available by the authors, without undue reservation.
